# Bridging the gap: ferroptosis of immune cells in the tumor microenvironment

**DOI:** 10.3389/fimmu.2025.1648432

**Published:** 2025-09-16

**Authors:** Weijing Wang, Huiyao Li, Shuai Liang, Yani Hu, Junli Ding, Xi Wu, Dong Hua

**Affiliations:** ^1^ Department of Oncology, The Affiliated Wuxi People's Hospital of Nanjing Medical University, Wuxi People's Hospital, Wuxi Medical Center, Nanjing Medical University, Wuxi, China; ^2^ The Affiliated Children's Hospital of Jiangnan University, Wuxi Children's Hospital, Wuxi School of Medicine, Jiangnan University, Wuxi, China

**Keywords:** ferroptosis, tumor microenvironment, immune cells, immunotherapy, nanomaterials

## Abstract

Ferroptosis, an iron-dependent form of regulated cell death driven by lipid peroxidation, is increasingly recognized as a pivotal immunomodulatory mechanism within the tumor microenvironment (TME). Beyond its well-established role in tumor cell elimination, emerging evidence reveals that immune cell subsets exhibit distinct susceptibility to ferroptosis, with profound consequences for antitumor immunity. This review systematically delineates the dual and cell-type-specific roles of ferroptosis across innate and adaptive immune populations: while ferroptosis-mediated depletion of immunosuppressive cells potentiates antitumor responses, immunostimulatory cells critically depend on ferroptosis defense pathways to sustain their survival and function—their dysfunction exacerbates immune evasion. We further decode the metabolic and signaling networks that govern immune cell ferroptosis and their dynamic interplay with immunotherapy and engineered nanomaterials. Finally, we critically addressed key challenges in clinical translation, including biomarker development, cell-specific delivery, and design of nanomaterials to minimize off-target effects. By elucidating the immune context-dependence of ferroptosis, this review provides a framework for developing precision therapies that harness ferroptosis-immune crosstalk to improve cancer therapy in the clinic.

## 1 Introduction

Tumor microenvironment (TME), a multifaceted system, is not limited to the immediate habitat of tumor cells themselves, diverse immune components but also is characterized by its dynamic complexity, which means it can promote the expansion and metastasis of tumors, as well as directly modulates their receptiveness to therapeutic interventions, while its effect is highly dependent on the host’s immune status. In detail, the anti-tumor immune system can be divided into two major parts: innate immunity and adaptive immunity, whereas the immune cells in the TME are stratified as immunosuppressive cells and immunostimulatory cells, and the balance between them determines the occurrence and development of cancer. More importantly, the quest to debilitate suppressive functionalities whilst fortifying stimulatory counterparts is still an urgent problem to be solved. At present, with the emergence of various forms of cell death, especially iron-dependent ferroptosis, has catalyzed a paradigm shift, presenting novel avenues for addressing this critical dilemma.

Ferroptosis is an iron-dependent programmed cell death mode, which is characterized by excessive Reactive Oxygen Species (ROS) accumulation and lipid peroxidation (1–3). Many metabolites produced by ferroptosis and various immune cells in the TME interact with each other. This interaction plays a dual role in innate and adaptive immune responses. For example, CD8^+^T cells, Natural Killer (NK) cells and Dendritic cells (DCs) are involved in antitumor immune responses, therefore, ferroptosis of these cells promotes immune evasion (4). On the contrary, M2 macrophages, regulatory T (Treg) cells, and myeloid-derived suppressor cells (MDSCs) inhibit antitumor immunity and promote the progression of cancer, therefore, the ferroptosis of these cells could enhance the effectiveness of immunotherapy (5, 6). The therapeutic exploitation of ferroptosis in the TME is complicated by its dichotomous effects on immune cells and their overlapping antioxidant defenses, requiring cell-selective approaches to simultaneously promote ferroptosis in immunosuppressive populations while protecting immunostimulatory cells. Selective cell-targeting strategies can balance the dual roles of ferroptosis in the TME by leveraging metabolic differences, surface markers, and microenvironment responses.

Moreover, the combination of ferroptosis with immunotherapy and nanomaterials has received substantial attention in recent years. On the one hand, Nano-carriers represent a novel type of nano-scale drug delivery system with the advantages of suitable size, easy modification, strong targeting ability, high cellular uptake, and good biocompatibility (7). Iron nanoparticles mediate Fenton reaction and the decomposition of hydrogen peroxide into highly active hydroxyl radicals. Nano-carriers have been successfully used in diagnosis and accurate treatment of drug delivery. On the other hand, regulating ferroptosis in combination with immunotherapy can help regulate the immune system and improve the anti-cancer effects of immunotherapy (8). Although the use of ferroptosis regulators in combination with immunotherapy or nanomaterials offers novel avenues for precisely control in specific immune cells, developing these combination strategies for clinical applications remains challenging.

Hence, in this review, we mainly focus on the ferroptosis of immune cells in the TME and discuss the precise potential of anti-cancer strategies involving the combination of ferroptosis in immunotherapy or nanomaterials, which could provide novel insights into the clinical diagnosis and treatment of cancer.

## 2 Ferroptosis

### 2.1 Mechanism of ferroptosis

**Figure 1:**
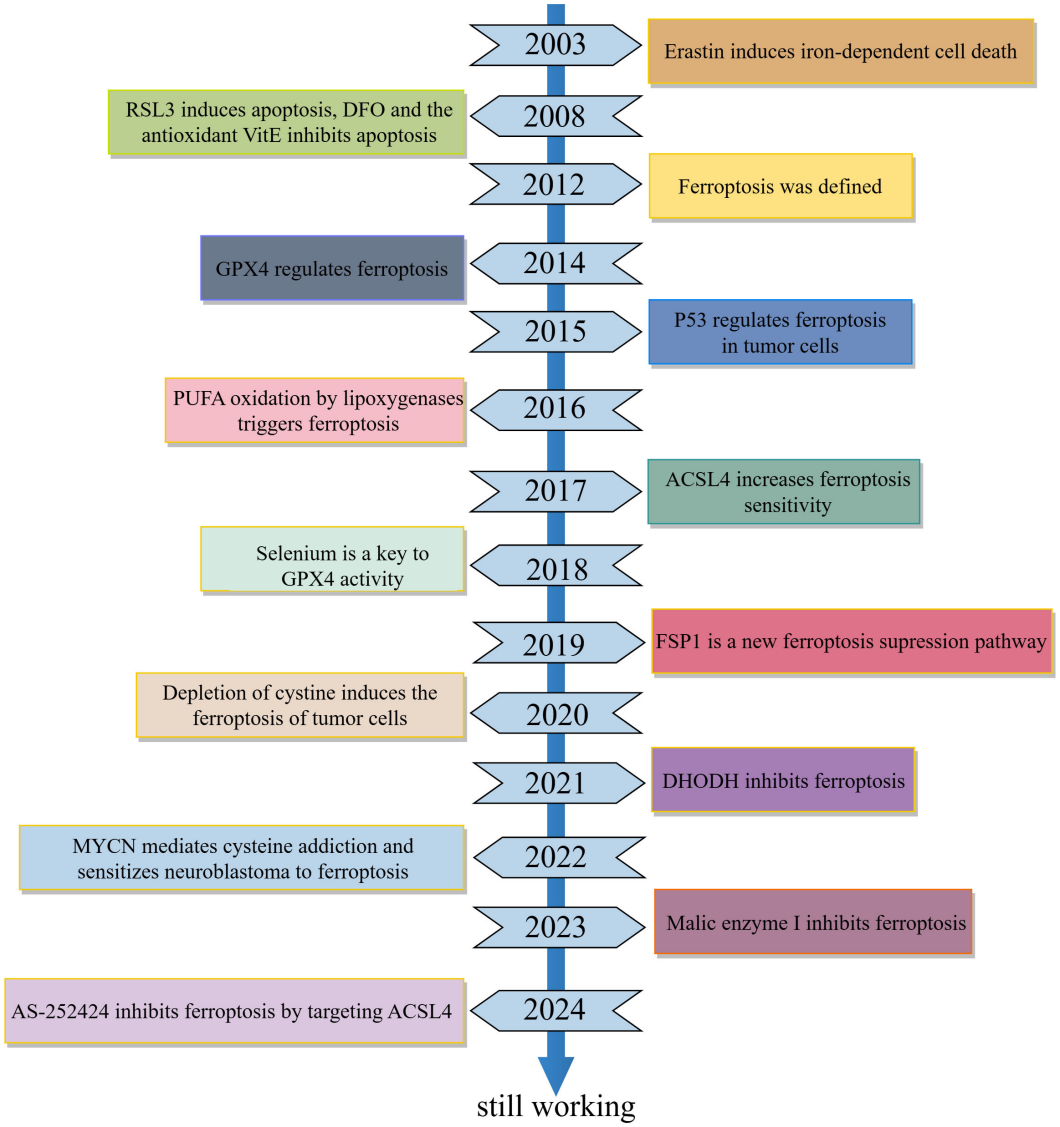
Significant Timeline for the Development of Ferroptosis. RSL3, RAS-selective lethal compound 3; DFO, Deferoxamine; VitE, Vitamin E; GPX4, Glutathione Peroxidase 4; PUFA, Polyunsaturated Fatty Acids; ACSL4, Acyl-CoA Synthetase Long Chain Family Member 4; FSP1, Ferroptosis Suppressor Protein 1; DHODH, Dihydroorotate Dehydrogenase; MYCN, MYCN Proto-Oncogene.

Ferroptosis, a recently discovered form of cell death, exhibits distinct morphological and biochemical characteristics (2) (**Figure 1**). Morphologically, ferroptosis is characterized by reduced mitochondrial volume, mitochondrial membrane shrinkage, an increased bilayer membrane density, and fewer or absent mitochondrial cristae, without nuclear condensation or chromatin margination (9). With regard to biochemical features, lipid-based ROS/Phospholipid hydroperoxides (PLOOHs) are the main factors leading to ferroptosis. Iron accumulation and lipid peroxidation, two key mechanisms of ferroptosis, cause oxidative damage to the cell membrane by promoting the production of PLOOHs (10). The accumulation of ROS and lipid peroxides during ferroptosis is promoted via dual mechanisms. On the one hand, the iron-dependent Fenton reaction catalyzes the generation of ROS. On the other hand, the activation of iron-dependent enzymes like lipoxygenases (LOXs) and cytochrome P450 oxidoreductase catalyze the production of PLOOHs and other oxidized lipid species, such as 1-steaoryl-2-15-HpETE-sn-glycero-3-phosphatidylethanolamine (SAPE-OOH) (3). Thus, cellular iron homeostasis and the metabolism of PLOOHs are crucial for the occurrence of ferroptosis.

### 2.2 Antioxidant pathways of ferroptosis

**Figure 2:**
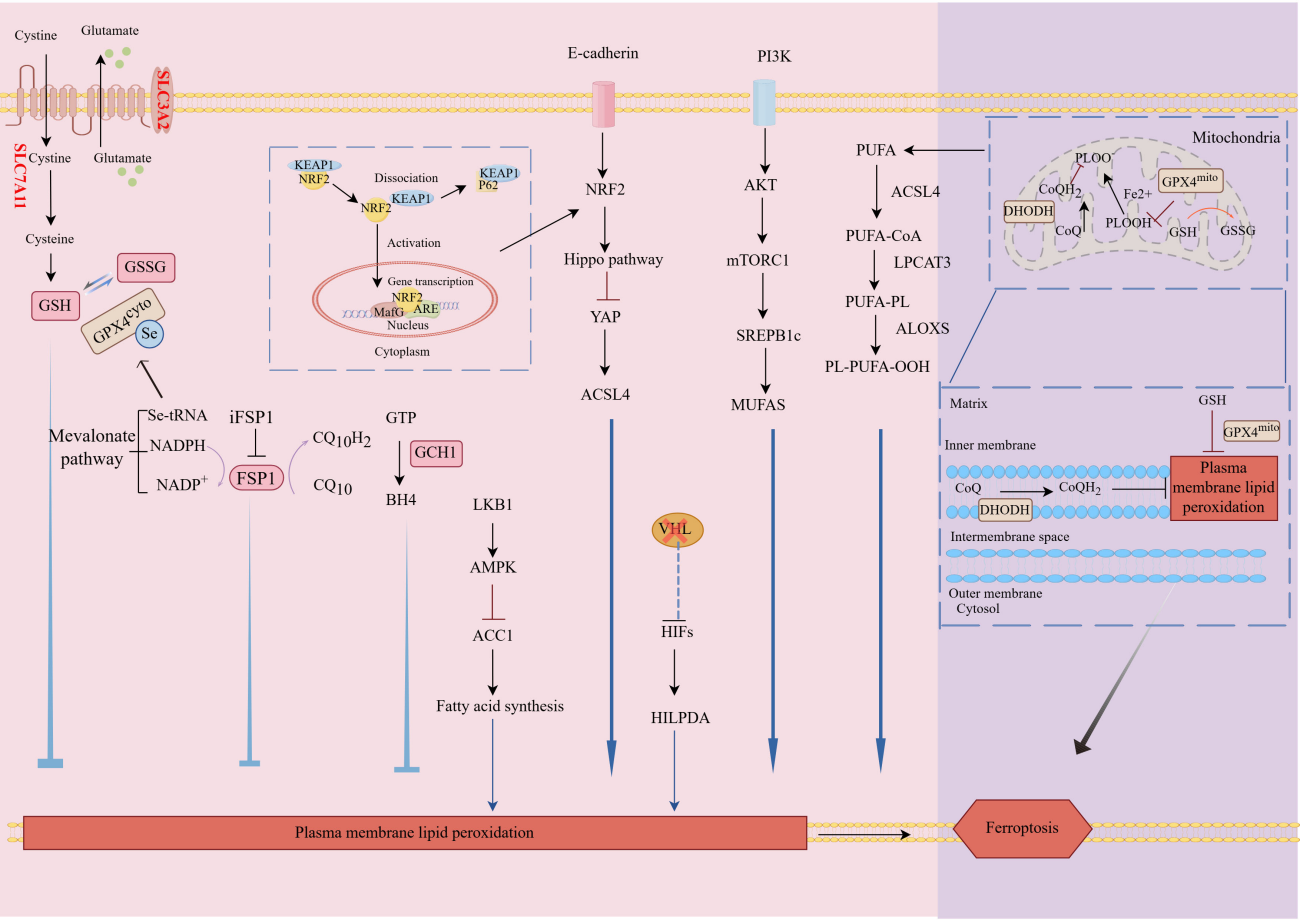
Mechanisms and regulatory pathways of ferroptosis. Ferroptosis is mainly caused by the peroxidation of PUFAs on specific membrane lipids. ACSL4 and LPCAT3 are crucial enzymes involved in the synthesis of PUFAs for membrane lipids. Hitherto, four regulatory pathways that reverse ferroptosis have been identified: the GPX4-GSH pathway, the FSP1-CoQH_2_ pathway, the GCH1-BH4 pathway, and the DHODH-CoQH_2_ pathway. Moreover, some signaling pathways including PI3K-AKT-mTOR pathway, LKB1-AMPK pathway, E-cadherin-Hippo-YAP/TAZ pathway, and VHL-HIF pathway also regulate ferroptosis through metabolic and stress-response mechanisms. PUFAs, Polyunsaturated Fatty Acids; ACSL4, Acyl-CoA Synthetase Long Chain Family Member 4; LPCAT3, Lysophosphatidylcholine Acyltransferase 3; GPX4, Glutathione Peroxidase 4; GSH, Glutathione; FSP1, Ferroptosis Suppressor Protein 1; CoQH_2_, Ubiquinol; BH4, Tetrahydrobiopterin; DHODH, Dihydroorotate Dehydrogenase; PI3K, Phosphoinositide 3-Kinase; AKT, Protein Kinase B; mTOR, Mechanistic Target of Rapamycin; LKB1, Liver Kinase B1; AMPK, AMP-activated Protein Kinase; E-cadherin, Epithelial Cadherin; Hippo, Hippo Signaling Pathway; YAP, Yes-associated Protein; TAZ, Transcriptional Co-activator with PDZ-binding Motif; VHL, Von Hippel-Lindau Tumor Suppressor Protein; HIF, Hypoxia-Inducible Factor; KEAP1, Kelch-like ECH-associated protein 1; NRF2, Nuclear factor erythroid 2-related factor 2; MafG, Musculoaponeurotic fibrosarcoma G; ARE, Antioxidant Response Element.

Ferroptosis can be inhibited by dual antioxidant pathways: Glutathione peroxidase 4 (GPX4)-dependent and non-GPX4-dependent pathways (**Figure 2**). Glutathione (GSH) metabolism plays a vital role in the GPX4-dependent antioxidant pathway, whereas CoQ regeneration serves as a cornerstone in non-GPX4 dependent antioxidant pathways (11).

#### 2.2.1 The canonical GPX4-regulated ferroptosis pathway

GPX4, a selenoprotein essential for cellular redox homeostasis, neutralizes PLOOHs on biological membranes by utilizing GSH as its obligate co-substrate (12). The cystine/glutamate transporter XC^-^, a heterodimeric complex composed of Solute Carrier Family 7 Member 11 (SLC7A11) and Solute Carrier Family 3 Member 2 (SLC3A2), mediates cystine uptake in exchange for intracellular glutamate efflux (1, 13). Notably, SLC7A11 serves as the central regulator of ferroptosis by maintaining redox homeostasis through GSH synthesis (14).Multiple regulatory mechanisms govern the expression of transporter XC^-^, encompassing targeted reduction of SLC7A11 levels, inhibition of SLC7A11 degradation and promotion of SLC7A11 expression, indicating that transporter XC^-^ can determine the susceptibility to ferroptosis (15, 16). Overall, both the biosynthesis/uptake of selenocysteine and the synthesis of GSH affect the homeostasis of GPX4, indicating that GPX4 plays a significant role in regulating ferroptosis (17, 18). Thus, the SLC7A11–GSH–GPX4 axis is believed to constitute the major cellular system defending against ferroptosis.

Paradoxically, upon GPX4 ablation, some cancer cell lineages remain resistant to ferroptosis revealing the existence of compensatory GPX4 independent defense mechanisms (19).

#### 2.2.2 GPX4-independent surveillance pathways

Emerging research has established the Ferroptosis inhibitor protein 1 (FSP1)- Ubiquinone (CoQ) axis as a parallel antioxidant system that functions as a compensatory mechanism specifically in GPX4-deficient cellular contexts (20). FSP1, originally characterized as apoptosis-inducing factor mitochondrial-associated protein 2 (AIFM2), has been redefined as a critical ferroptosis inhibitor through its N-terminal myristoylation-mediated membrane targeting. This membrane-localized oxidoreductase enzymatically reduces CoQ10, a mevalonate pathway-derived metabolity, to its reduced form ubiquinol. Apart from its well-known function in mitochondrial electron transport, it has been proposed that FSP1 exerts its potent anti-ferroptosis activity through generating the extra-mitochondrial CoQH2 pool as radical-trapping antioxidants (21, 22). The sources of the extra-mitochondrial CoQ harnessed by FSP1 for ferroptosis defense remain to be established.

Recent studies have positioned GTP cyclohydroxylase-1 (GCH1) as another critical regulator of ferroptosis (23, 24). Tetrahydrobiopterin (BH4), an essential redox cofactor, functions canonically in aromatic amino acid hydroxylation and nitric oxide synthase activity. Additionally, it serves as a critical mediator of ferroptosis resistance by catalyzing the rate-limiting step in the BH4 metabolic pathway (25). It was proposed that GCH1 confers ferroptosis resistance through the biosynthesis of BH4, which acts as a radical-trapping antioxidant, as well as through GCH1-mediated production of CoQH2 and Phospholipids (PLs) containing two Polyunsaturated Fatty Acid (PUFA) tails (23). The subcellular compartment wherein the GCH1–BH4 system operates needs to be investigated.

A recent study uncovered a mitochondria-localized antioxidant system mediated by Dihydroorotate Dehydrogenase (DHODH) capable of compensating for GPX4 deficiency by neutralizing lipid peroxidation within mitochondria (26). DHODH, a pyrimidine biosynthesis enzyme in the mitochondrial inner membrane, reduces CoQ to CoQH2. Therefore, DHODH functions as mitochondrial antioxidant system that protects against oxidative damage.

In addition, the crosstalk between ferroptosis and other signaling pathways has only been extensively studied in cancer. Key pathways such as PI3K-AKT-mTOR, LKB1-AMPK, E-cadherin-Hippo-YAP/TAZ, and VHL-HIF critically modulate ferroptosis in cancers via metabolic regulation and stress-response mechanisms (27).

In the future, integrating strategies to target ferroptosis defense pathways and enhance the efficacy of cancer immunotherapy will represent a promising therapeutic approach.

## 3 The ferroptosis of immune cells

**Figure 3:**
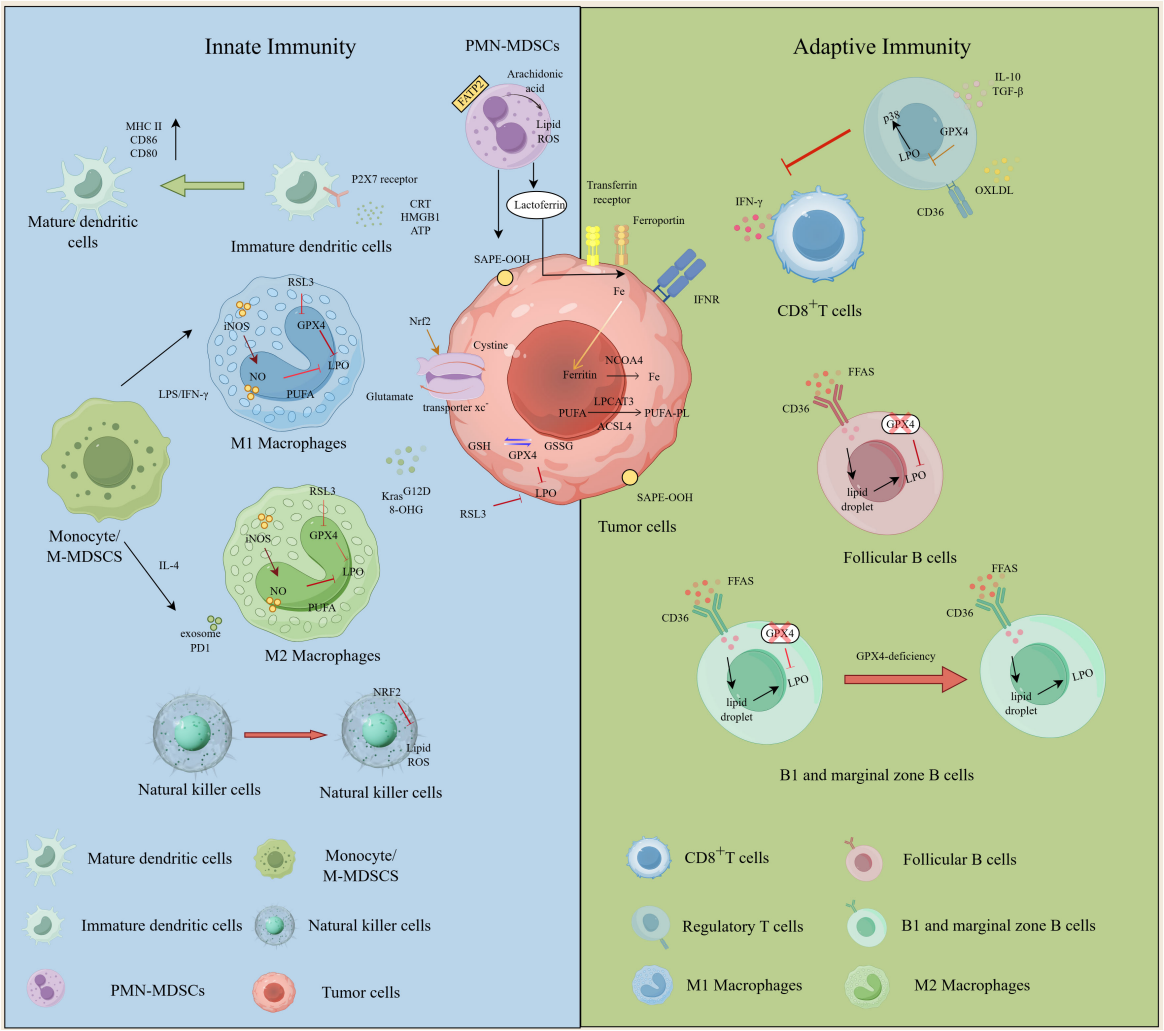
Regulatory mechanisms of ferroptosis in various immune cells. The figure depicts different regulation of ferroptosis in immune inhibitory cells and immune stimulating cells and its different function in innate immunity and adaptive immunity. In innate immunity, M1 and M2 macrophages show disparate sensitivity to ferroptosis. Mature DC cells express higher levels of CD86, CD80, MCHII. Ferroptosis in PMN-MDSCs plays an immunosuppressive role, which is mediated through the release of oxidized lipids and promoted by FATP2.Phosphatidylethanolamine widely exists on the ferroptosis of cell membranes, especially SAPE-OOH. Tregs play an important role in maintaining immune tolerance. Activating the function of NRF2 in NK cells can reverse the inhibitory effect on NK cells in the TME. In adaptive immunity, CD8^+^T cells release IFN-γ which can be suppressed by p38.Tregs suppress immune response and promote tumor immune escape by secreting cytokines such as IL-10 and TGF-β.GPX4-deficient B1 and MZ B cells are more sensitive to ferroptosis because of the higher expression of the fatty acid transporter CD36 while GPX4-deficient follicular (FO) B cells are less susceptible to ferroptosis due to reduced expression of intracellular fatty acids. DC, dendritic cell; FO B cell, follicular B cell; iNOS, inducible nitric oxidesynthase; M-MDSC, monocytic myeloid-derived suppressor cell; PMN-MDSC, polymorphonuclear myeloid-derived suppressor cell; MZ B cell, marginal zone B cell; NK, natural killer; ROS, reactive oxygen species; Treg, regulatory T cell; SAPE-OOH,1-steaoryl-2-15-HpETE-sn-glycero-3-phosphatidylethanolamine;NRF2, Nuclear factor erythroid 2-related factor2; TME, tumor microenvironment.

The TME comprises a complex network of tumor cells, immune cells, and stromal components, where ferroptosis acts as a dual-edged sword—directly eliminating tumor cells while indirectly shaping immune responses. When tumor cells undergo ferroptosis, they release damage-associated molecular patterns (DAMPs) such as high mobility group box 1 (HMGB1), adenosine triphosphate (ATP), and KRASG12D, which engage pattern recognition receptors (e.g., toll-like receptor 4,TLR4; P2X Ligand-Gated Ion Channel 7,P2X7) on innate immune cells to trigger immunogenic cell death (ICD) or modulate polarization states (e.g., STAT3-driven M2 macrophage skewing via AGER/RAGE) (28–32). However, immune cells themselves exhibit cell-type-specific susceptibility to ferroptosis, governed by distinct metabolic and antioxidant pathways (**Figure 3**). Emerging evidence reveals a dichotomy: studies have shown that macrophages, CD8+T cells, B cells and Treg cells tend to resist ferroptosis through the canonical GPX4-regulated ferroptosis pathway and neutrophils, DCs and NK cells prefer the GPX4-independent surveillance pathways (33–36). Therefore, we dissect these mechanisms across immune subsets and discuss therapeutic strategies to exploit ferroptosis-immune crosstalk.

### 3.1 Innate immunity

#### 3.1.1 Dendritic cells

Ferroptosis of early tumor cells can promote the maturation and activation of DCs, enhance the antigen-presenting function of DCs, and stimulate effector T cells to exert anti-tumor effects (37). However, this beneficial effect appears to be context-dependent, as Wiernicki found that during the maturation of bone marrow-derived DCs (BMDCs), the expression of 12/15-LOX and the production of lipid peroxides increased, reversing cell maturations (38). This paradoxical finding suggests that ferroptosis may have dual roles in DC biology depending on cellular context and timing.

Indeed, ferroptosis can impair DCs antigen presentation through multiple mechanisms. Early lipid ROS accumulation downregulates CD86, CD40, and MHC II expression while reducing Interleukin-12(IL-12) and Interferon-γ(IFN-γ) secretion, impairing T cell activation (37). Beyond maturation inhibition, disrupted phosphatidylserine exposure and delayed calreticulin translocation (synchronized with membrane rupture) reduce phagocytic efficiency. The metabolic consequences are equally significant, with Oxidized Phospholipids (oxPL) uptake inducing lipid droplet accumulation while Acyl-CoA Synthetase Long-Chain Family Member 4 (ACSL4)-mediated membrane remodeling destabilizes immune synapses. These changes are compounded by transcriptional reprogramming, where coordinated NF-κB/STAT4/CCR7 downregulation and CCL3/4 upregulation, combined with ROS-induced mitochondrial/Endoplasmic Reticulum(ER) damage, collectively suppress antigen processing and MHC presentation (37, 39).

The progression of ferroptosis in DCs follows a distinct temporal pattern. Initially, lipid ROS accumulation impairs DC maturation and phagocytic capacity. As the process continues, transient calreticulin (CRT) exposure and ATP release occur during an intermediate phase, though immunogenicity remains limited. Ultimately, terminal membrane rupture releases DAMPs (High Mobility Group Box 1, HMGB1, Lactate Dehydrogenase, LDH) and cytokines (Tumor Necrosis Factor, TNF, IFN-β), while permanently impairing cross-presentation capability. This phased deterioration of DC function helps explain why the timing of ferroptosis induction is so critical for immune outcomes (37).

This timing dependence is clearly demonstrated by Efimova’s team, who showed that the GPX4 inhibitor RAS-Selective Lethal compound 3 (RSL3) stimulated ICD in tumor cells to activate DCs and induce anti-tumor immunity in an animal tumor model (40). Their work revealed that while early ferroptotic tumor cells (1 h post-RSL3) induced BMDC maturation, late ferroptotic cells (24 h post-RSL3) were simply phagocytosed without immunostimulatory effects. Importantly, the anti-tumor protection conferred by early ferroptotic cells depended on ATP-Purinergic Receptor P2X7 signaling, as blockade with oxidized-ATP abolished this effect, highlighting how DCs process and present tumor antigens via MHC complexes to activate T cells (41).

While DCs can enhance anti-tumor immunity through multiple mechanisms including NK cell activation, their function is particularly vulnerable in the tumor microenvironment. Tumor-infiltrating DCs, which tend to accumulate more lipids, show increased susceptibility to ferroptosis and consequent antigen presentation failure (42, 43). This vulnerability can be modulated, RSL3-induced DC dysfunction is reversible with Peroxisome Proliferator-Activated Receptor Gamma (PPARG) knockout, while Sestrin2 protects against Lipopolysaccharide (LPS)-induced ferroptosis (44). The accumulation of 4-Hydroxynonenal (4-HNE)-protein complexes in tumor-associated DCs further illustrates how oxidative stress can trigger DCs dysfunction through X-box Binding Protein 1 (XBP1) activation (45). Together, these findings paint a complex picture where ferroptosis-related molecules critically regulate DCs maturation and function, with important implications for both tumor immunology and therapeutic development.

#### 3.1.2 Natural killer cells

NK cells, which are considered inherently immunized cells, are involved in the perforin/granzyme pathway, Factor Associated Suicide (Fas) and Factor Associated Suicide Ligand (FasL) interactions, TNF-α and TNFR-1 interactions, and antibody-dependent cytotoxicity (46). As they play an important role in anti-tumor immunity, their functional inhibition can promote tumor growth. It was found that the tumor-infiltrating NK cells contain higher levels of ferroptosis and lipid oxidation-related proteins, and the cell morphology was typical of ferroptosis (47). They have higher oxidative stress levels and weaker glucose metabolism, which eventually results in their dysfunction in the TME. Nuclear factor erythroid 2–related factor 2 (NRF2) helps NK cells regain anti-tumor activity in the TME (48). Therefore, ferroptosis of NK cells leads to insufficient anti-tumor immune function, which prevents the elimination and inhibition of tumor cells in a timely manner and helps tumor cells evade immune surveillance.

#### 3.1.3 PMN-MDSCs

Neutrophils, originating from the bone marrow, are the first line of defense against microbial infection (49). In addition to other myeloid cells, neutrophils have been extensively investigated for their important role in cancer. Neutrophils have been shown to exert direct and indirect anti-tumor effects during the initiation of tumorigenesis and the early stages of tumor growth (50). Ly6G^+^ neutrophils inhibit B cell ferroptosis by secreting IL-6, which binds to the IL-6R on B cells and activates the downstream STAT3/SLC7A11 pathway. Molecules such as B-cell activating factor (BAFF) and A Proliferation-Inducing Ligand (APRIL) may also participate in the interaction between neutrophils and B cells, but IL-6 is the dominant signal. Treatment with anti-IL-6 antibodies significantly increases ferroptosis markers in B cells and reduces the GSH/Glutathione Disulfide (GSSG) ratio (51).

However, tumor-derived signals can alter bone marrow formation, leading to the expansion and pathological activation of neutrophils, called polymorphonuclear MDSCs (PMN-MDSCs) (52).

PMN-MDSCs play an immunosuppressive role by attenuating immune responses mediated by T cells, B cells, and NK cells. PMN-MDSCs employ three distinct but interconnected mechanisms to mediate immunosuppression. First, through metabolic regulation, their lipid peroxidation products (4-HNE and Malondialdehyde, MDA) directly activate arginase-1 (ARG1) and inducible nitric oxide synthase (iNOS), effectively suppressing T cell function (53). Second, by secreting inflammatory mediators, they upregulate Prostaglandin E2 (PGE2) biosynthesis genes, leading to enhanced PGE2 release that simultaneously inhibits T cell proliferation/function (54) and promotes the production of immunosuppressive cytokines IL-10 and transforming growth factor-β(TGF-β) (55). Finally, they exert direct cytotoxicity by impairing T cell antigen responsiveness through ROS generation and inducing Cytotoxic T cells (CTL) apoptosis via the Fas/FasL pathway (56).

Ferroptosis-related genes have been associated with genes found in PMN-MDSCs in various cancer types (54). A study analyzed liver metastases and adjacent normal tissues from a 63-year-old male Colorectal Cancer patient. Tissues were digested into single-cell suspensions, then processed by flow sorting and single-cell RNA sequencing, which revealed a significant increase in the death proportion of neutrophils. Additionally, pathways linked to ferroptosis, iron metabolism and glutathione metabolism were notably enriched in these granulocytes, providing the first evidence of immune cell ferroptosis in the TME (57). Previous studies suggested that N-Acylsphingosine Amidohydrolase 2 (ASAH2) is highly expressed in MDSC cells. Its inhibition enhances the stability of the p53 protein and upregulates Heme Oxygenase 1 (Hmox1), thereby inhibiting the production of lipid ROS and preventing the ferroptosis of MDSCs (58). In immunized mice, inhibition of ferroptosis through genetic and pharmacological means has been shown to counteract the immunosuppressive effects of PMN-MDSCs, inhibit tumor progression, and cooperate with immune checkpoint blockers (ICBs) to inhibit tumor growth (59).

Tumor-infiltrating PMN-MDSCs exhibit complex dual functions in anti-tumor immunity through ferroptosis-dependent mechanisms. These cells can promote tumor cell ferroptosis (particularly in glioblastoma), inducing tumor death and growth inhibition. Conversely, when PMN-MDSCs themselves undergo ferroptosis in the tumor microenvironment, they release lipid oxides via fatty acid transporter (FATP2)-mediated pathways, creating an immunosuppressive niche that paradoxically supports tumor progression. This immunosuppression involves the secretion of oxidized phospholipids and immunomodulatory factors that activate ARG1 and iNOS to suppress T cell proliferation (53). However, some studies have indicated that ferroptosis may suppress (rather than enhance) the immunosuppressive function of PMN-MDSCs (54). Notably, the net immunological impact appears context-dependent: while hepatocellular carcinoma models show that ferroptosis inhibition abolishes PMN-MDSC-mediated T cell suppression and synergizes with checkpoint inhibitors (60), non-small cell lung cancer studies demonstrate that ferroptosis inducers can simultaneously reduce MDSC accumulation and enhance T cell function (61). These divergent outcomes highlight tumor-type specific regulation of PMN-MDSC biology by ferroptosis, where the balance between direct tumoricidal effects and indirect immune modulation determines the ultimate therapeutic outcome.

A comprehensive elucidation of the mechanistic interplay between PMN-MDSCs and ferroptosis, particularly regarding their tumor-type-specific regulation of immune suppression versus activation, represents a crucial unmet need in the field.

#### 3.1.4 Tumor-associated macrophages

Tumor-associated macrophages (TAMs) are the main immune cells in the TME, and a high degree of TAM infiltration often indicates a poor prognosis (62). Monocytes and mononuclear MDSCs (M-MDSCs) exhibit the same classical and pathologically activation patterns as the aforementioned neutrophils and PMN-MDSCs, respectively. They promote anti-tumor immunity by activating T cells and TAMs. Studies have shown that TAMs derived from M-MDSCs retain their immunosuppressive activity when compared with those derived from classical monocytes (63).M-MDSCs derived TAMs maintain immunosuppressive activity mainly by secreting various mediators, which promote angiogenesis and metastasis and facilitate tumor progression (64).

##### 3.1.4.1 Ferroptosis susceptibility in M1/M2 macrophages

TAMs exhibit high plasticity, whereas the development and homeostasis of resident macrophages in lung, peritoneum, spleen and bone marrow (which can be regarded as M0 state) are not affected (62). LPS/IFN-γ stimulation can activate the polarization of M0 macrophages to the M1 macrophages, which promotes inflammation and inhibits tumor growth. IL-4 stimulation can activate the polarization of M0 macrophages to the M2 macrophages, which suppresses inflammation and promotes tumor growth.

In addition, CD44 is a key molecule linking ferroptosis and macrophage polarization. By regulating cell adhesion and ROS generation, it promotes the recruitment of M1 macrophages. Anti-CD44 antibodies can reduce M1 accumulation and suppress ferroptosis. Enhancer of Zeste Homolog 2 (EZH2), through epigenetic modifications, inhibits pro-inflammatory genes and may promote M2 polarization. Peroxisome Proliferator-Activated Receptor Alpha (PPARα) may indirectly reduce macrophage inflammatory responses by suppressing lipid peroxidation upon activation (65).

The sensitivity of these two macrophage subtypes to ferroptosis is different. In most tumors, M2 macrophages express ARG1 and CD206 while secreting IL-10 and TGF-β, thereby promoting angiogenesis, matrix remodeling, and immunosuppression to maintain tumor cell growth. On the contrary, M1 macrophages express CD86 and iNOS, exerting tumor-suppressive effects by producing IL-1α/β, IL-6, TNF-α, and ROS (66). M2 macrophages are more susceptible to ferroptosis owing to the lower expression of iNOS and the production of less NO free radicals. NO radicals react with lipid free radicals to suppress intracellular lipid peroxidation and inhibit the occurrence of ferroptosis (6). Given that M1 macrophages produce more NO radicals, which inhibit 15- LOX catalyzed PUFA peroxidation, thereby being resistant to ferroptosis.

##### 3.1.4.2 Therapeutic implications of macrophage ferroptosis

Ferroptosis inducers (such as the GPX4 inhibitor RSL3) can lead to the elimination of M2 macrophages, but do not affect M1 macrophages, resulting in synergistic anti-tumor effects. Given that M1 macrophages have antitumor activity, it is necessary to focus on the transformation of M2 macrophages into the M1 phenotype in the context of ferroptosis. Studies have demonstrated that ferumoxytol, an iron oxide nanoparticle formulation, (the detailed mechanism of Fenton reaction in Section 4.2) modulates intracellular iron homeostasis and induces M1 macrophage polarization, ultimately triggering ferroptosis (67).

Shi and Hu developed iron-doped mesoporous silica nanoparticles loaded with citrate and dextran (DFHC) (68), which, similar to the FePt@MnO@DSPE-PEG5000-FA system described in Section 4.2, induces ferroptosis by modulating iron metabolism in TAMs. Nanomaterials regulate the ferroptosis sensitivity of macrophages by modulating iron metabolism, inducing lipid peroxidation, or delivering ferroptosis inducers (e.g., RSL3, iFSP1) in a targeted manner. For specific design strategies of nanomaterials, we will further discussed in detail in Section 4.2. In addition, a study reported the development of the albiziabioside A–DCA (AlbA-DCA) conjugate to inhibit breast cancer cell proliferation (69). Mechanistically, this conjugate inhibited GPX4, a key ferroptosis-related gene, and the activity of M2 macrophages to induce ferroptosis of breast cancer cells.

Furthermore, the monocyte-phagocyte system is not sensitive to ferroptosis in some cases. For instance, human peripheral blood mononuclear cells (PBMCs) are resistant to ferroptosis induced by erastin. On the contrary, erastin promotes the proliferation and differentiation of human PBMCs into B cells and NK cells by regulating bone morphogenetic protein (BMP), thereby enhancing anti-tumor immunity (70). Similarly, macrophages harboring the p53P47S mutation are resistant to ferroptosis (71). Recent studies have shown that when ferroptotic PMN-MDSCs in the TME induce ferroptosis of TAMs, the ferroptotic TAMs exert strong tumor-promoting effects. However, loss of myeloperoxidase (MPO) and 12/15-Arachidonate Lipoxygenase (ALOX) in PMN-MDSCs significantly inhibits these tumor-promoting effects (54).

The evidence demonstrates that pharmacological modulation of macrophage polarization toward the M1 phenotype by targeting ferroptosis sensitivity or epigenetic reprogramming represents a promising approach to enhancing tumoricidal immunity.

### 3.2 Adaptive immunity

#### 3.2.1 CD8^+^T cells

##### 3.2.1.1 Ferroptosis promotes CD8^+^ T cells depletion

CTLs, also known as CD8^+^ T cells, are the primary executors of adaptive immunity. Ferroptosis regulates the infiltration of CD8^+^ T cells in the TME, thereby affecting their recruitment and anti-tumor immune function. Depletion of CD8^+^ T cells is associated with an increase in lipid uptake and intracellular lipid peroxidation (72, 73). Liao et al. further demonstrated that IFN-γ released by CD8^+^ T cells synergizes with arachidonic acid to effectively induce ferroptosis across multiple cancer types (74). Recently, Cui’s team revealed a novel immunosuppressive mechanism in cancer (75), the TME produces a large amount of oxidized low-density lipoprotein (Ox-LDL), which is internalized by CD8^+^ T cells with high expression of CD36 (a fatty acid transport molecule). Upregulation of intracellular lipid peroxidation triggers the activation of p38, a stress-reactive protein, and T cell ferroptosis, leading to downregulation of IFN-γ and TNF-α, which results in the dysfunction of CD8^+^ T cells and tumor immune escape. The expression of CD36 is positively correlated with the expression of PD-1 and TIM-3. Moreover, DEP Domain Containing 5 (DEPDC5) regulates the homeostasis of CD8^+^ T cells in peripheral blood, protecting them from ROS induced ferroptosis and exerting anti-tumor effects (76). Consequently, blocking CD36 or maintaining DEPDC5 can inhibit ferroptosis in CD8^+^ T cells and restore their anti-tumor immune function.

In summary, ferroptosis-related factors interact with cytotoxic T lymphocytes (CTLs), modulating their activity and function, while CTLs reciprocally influence ferroptosis levels in tumor cells.

##### 3.2.1.2 CD8^+^ T cells promote ferroptosis in tumor cells

IFN-γ downregulates the expression of SLC3A2 and SLC7A11 on tumor cell surfaces through the JAK-STAT1 pathway (34). This negative regulation reduces the intracellular transport of cystine and the production of intracellular glutathione, resulting in excessive ROS accumulation, tumor cell ferroptosis, and the release of DAMPs. DAMPs, in turn, increase CD8^+^ T cells infiltration in tumors and improve the anti-tumor effects of immunotherapy. Phospholipid profiling revealed that arachidonic acid is preferentially incorporated into phosphatidylethanolamine (PE) and phosphatidylcholine (PC) species containing C16 and C18 acyl chains in an ACSL4-dependent manner (77). Palmitoleic acid and oleic acid, two common C16 and C18 fatty acids in circulation, enhance the abundance of arachidonic acid (d5)-bound PE and PC in tumor cells. Notably, Lysophosphatidylcholine Acyltransferase 3 (LPCAT3) and LOX are respectively involved in the incorporation of arachidonic acid into membrane phospholipids and the oxidation of these phospholipids, collectively promoting ACSL4-dependent tumor ferroptosis induced by IFN-γ and arachidonic acid (78). For example, compared with wild-type mice, ACSL4-knockout mice exhibited reduced CD8^+^ T cell infiltration in tumors, decreased IFN-γ and TNF-α expression, lower tumor cell lipid peroxide levels, and accelerated tumor growth (74). Altogether, these findings suggest that inducing the ferroptosis of tumor cells can improve anti-tumor immunity and enhance the efficacy of immunotherapy.

CD8^+^ T cells exhibit seemingly paradoxical roles in ferroptosis regulation: they promote tumor cell ferroptosis while remaining vulnerable to ferroptosis themselves. This delicate balance is jointly regulated by T cell activation status and TME metabolic characteristics. Naive/memory T cells, being metabolically quiescent and reliant on mitochondrial oxidative phosphorylation (OXPHOS) with strong antioxidant capacity, are resistant to ferroptosis. In contrast, effector T cells with high glycolysis and ROS production are susceptible to ferroptosis. Exhausted T cells face exacerbated ferroptosis as PD-1 signaling further suppresses antioxidant pathways (e.g., NRF2) (79). Glutamine-addicted effector T cells become more susceptible to ferroptosis under glutamine-deprived conditions in the tumor microenvironment due to impaired GSH synthesis (80). GSH plays a crucial role in maintaining regulatory T cell function by restricting serine metabolism (81).

Notably, the sensitivity of tumor cells and CTLs to ferroptosis in the TME remains controversial. Some studies suggest ferroptosis inducers selectively target tumor cells without compromising CTL function, thereby enhancing immunotherapy efficacy (82). Conversely, other evidence shows GPX4 inhibitors can trigger CTL ferroptosis *in vitro*, impairing their anti-tumor activity and promoting immune escape (83, 84). This cell type-specific susceptibility may stem from metabolic heterogeneity: metabolically, CD8^+^ T cell function is intricately regulated by the interplay of lipid, glucose, and amino acid metabolism. When facing amino acid or glucose limitation, the GAP Activity Toward Rags 1 (GATOR1) complex acts as a metabolic sensor to suppress mTOR complex 1 (mTORC1) activation. This mTORC1-dependent metabolic reprogramming produces numerous biochemical intermediates that are mechanistically connected to ferroptosis pathways (76). Thus, the specificity of ferroptosis inducers and their mechanistic underpinnings demand systematic investigation to reconcile these disparities. Future investigations employing single-cell metabolomics approaches will be essential to delineate subset-specific metabolic signatures and optimize precision therapeutic strategies.

#### 3.2.2 Regulatory T cells

Treg cells are key inhibitory cells that maintain immune tolerance and escape of tumor cells. They are a subset of CD4^+^ T cells with immunosuppressive function and are composed of thymus-derived natural Treg cells and peripherally induced Treg cells. In the TME, Treg cells suppress the immune response and promote tumor immune escape by secreting cytokines such as IL-10 and TGF-β (85, 86). Treg cells deliver inhibitory signals through direct contact with antigen presenting cells or effector T cells via surface molecules (Cytotoxic T-Lymphocyte-Associated Protein 4, CTLA-4; PD-1). This contact-dependent suppression requires intercellular communication (e.g., cAMP transfer through gap junctions). During ferroptosis, Treg cells exhibit elevated mitochondrial superoxide (mitoSOX), disrupting OXPHOS and Fatty Acid Oxidation (FAO) - critical metabolic pathways that maintain Treg cells survival and suppressive function (35).Under the co-stimulation of the T-cell receptor/CD28, knockout of GPX4 in Treg cells increases the level of lipid oxidation and ferroptosis, resulting in the ferroptosis of Treg cells and suppression of their immunoregulatory function. Treg cells with GPX4 knockout can enhance TH17 cell-mediated inflammatory responses by secreting IL-1β. Furthermore, they can activate the function of DCs and CD8^+^ T cells, potentiating anti-tumor immune responses. Specific knockout of GPX4 in Treg cells has been shown to inhibit the growth of MC38 colon cancer and B16F10 melanoma xenografts in animal models (87).

Treg cells shape the immunosuppressive TME through multiple mechanisms, with their metabolic vulnerabilities (particularly ferroptosis sensitivity) offering a critical breakthrough for reversing tumor immune escape. Targeted modulation of Treg cell survival and function holds promise for reprogramming the TME from immunosuppressive to immunostimulatory states, thereby opening new avenues for cancer immunotherapy.

#### 3.2.3 B cells

B cells produce and release antibodies against specific antigens, playing an important role in humoral immune responses (88). Upon activation, conventional B cells (usually called B2 cells) differentiate into plasma and memory B cells. Other B cell subtypes include B1 cells, marginal zone (MZ) B cells, follicular B cells, and regulatory B (Breg) cells. Follicular B cells are the largest group of B cells and are mainly involved in humoral immunity.

Different subtypes of B cells exhibit varying sensitivity to ferroptosis (89). A recent study showed that GPX4 is not required for the development and immune function of follicular B cells. However, it is required for both processes in B1 and MZ B cells because the loss of GPX4 expression in B1 and MZ B cells promotes lipid peroxidation and ferroptosis. Moreover, compared with follicular B cells, B1 and MZ B cells have higher expression of the fatty acid transporter CD36, which enhances their ability to internalize fatty acids.

Following CD36 binding to PUFAs, APT1-mediated depalmitoylation promotes the formation of a signaling complex between CD36 and spleen tyrosine kinase, triggering caveolae-mediated endocytosis. This process significantly increases intracellular PUFA accumulation, leading to a “lipid peroxidation storm” under GPX4-deficient conditions. While B1/MZ B cells can activate parallel antioxidant systems through exogenous CoQ10 supplementation or FSP1 overexpression, this pathway exhibits limited efficacy in follicular B cells (22). Concurrently, DAMPs such as HMGB1 released from ferroptotic B1/MZ B cells promote DC maturation via the TLR4/Myeloid differentiation primary response 88(MyD88) pathway while inducing IFN-γ secretion. IFN-γ inhibits GSH synthesis by downregulating SLC7A11, establishing a pro-ferroptotic positive feedback loop. Nuclear Receptor Coactivator 4(NCOA4)-mediated ferritinophagy increases free iron levels, which not only enhances Fenton reactions but also upregulates Transferrin Receptor 1(TfR1) expression through iron-responsive element (IRE) activation, further exacerbating iron overload (90). Follicular B cells maintain high IL-10 expression, which preserves reduced glutathione pools through STAT3 signaling while suppressing NADPH Oxidase 2(NOX2) activity, creating a dual antioxidant defense system (91). As an immunosuppressive cytokine, IL-10 inhibits the activity of T cells and NK cells to modulate immune responses and maintain immune homeostasis. Future studies should investigate combined CD36 inhibition and PD-L1 targeting strategies to protect B1 and MZ B cells from ferroptosis.

Altogether, ferroptosis is involved in the survival and potential anti-cancer effects of B cells. However, the specific underlying mechanisms warrant further investigation.

## 4 Ferroptosis in combination with immunotherapy and nanomaterials

### 4.1 Ferroptosis in combination with immunotherapy

Immunotherapy is a recently developed anti-cancer strategy, following surgery, chemotherapy, and radiotherapy. Ferroptosis of tumor cells can enhance the anti-tumor responses of immune cells in the TME. Therefore, combining the induction of tumor cell ferroptosis with immunotherapy presents significant potential for clinical application in cancer therapy (**Table 1**).

**Table 1:** Selected ferroptosis-inducing compounds/drugs in preclinical and clinical development.

Compound/Drug	Caner type	Target	Function	Type of test	Reference
Gemcitabine	Pancreatic cancer	GPX4	Inhibit the level of GPX4	Preclinical Research	(92)
Etoposide	Myelogenous leukemia	GPX4	Inhibit the level of GPX4	Preclinical Research	(93)
Sorafenib	Hepatocellular carcinoma	SLC7A11	Reduce cysteine uptake	Preclinical Research	(94)
Cisplatin	Ovarian cancer, lung cancer, thyroid cancer, lymphosarcoma,	GSH	Reduce GSH	Preclinical Research	(95)
Siramesine, Lapatinib	Breast cancer	Fe	Increase the intracellular iron level	Preclinical Research	(96, 97)
Decitabine	Myelodysplastic syndrome	GPX4, GSH	Decreases GSH levels and inhibits GPX4 activity	Preclinical Research	(98)
Sulfasalazine	Head neck cancer, Glioblastoma, Fibrosarcoma	SLC7A11	Inhibit transporter XC^-^	Phase I clinical trial **NCT04205357**	(99–101)
Altretamine	Diffuse large B cell lymphoma	GPX4	Inhibit the level of GPX4	Preclinical Research	(102)
Statins	Lung cancer	GPX4	Inhibit the level of GPX4	Preclinical Research	(103)
Vorinostat	Lung cancer	SLC7A11	Inhibit SLC7A11 expression	Preclinical Research	(104)
Olaparib	Ovarian cancer	SLC7A11	Suppress SLC7A11 mediated GSH synthesis	Preclinical Research	(105)
Temozolomide	Glioblastoma	GSH	Reduce GSH	Phase II clinical trial **NCT06218524**	(106)

NRF2, Nuclear factor erythroid 2-related factor 2; GSH, Glutathione; HO-1, Heme Oxygenase-1; ROS, Reactive Oxygen Species; GPX4, Glutathione peroxidase 4; SLC7A11, Solute carrier family 7 membrane 11. The bolded NCT04205357 and NCT04205357 represent the ongoing clinical trials mentioned in the main text.

Programmed deathligand1 (PD-L1) is highly expressed in most tumor tissues and is closely related to the development of malignant tumors (107). Wang’s team found that treatment with PD-L1 inhibitor significantly decreased tumor volume and counteracted the immunosuppressive effects of PD-L1 (34). In ID8 and B16 subcutaneous tumor-bearing mouse models treated with PD-L1 inhibitor every three days, lipid ROS levels were significantly elevated in the anti-PD-L1 group. However, subsequent treatment with the ferroptosis inhibitor liproxstatin-1 restored the immunosuppressive effects of PD-L1. These findings suggest that ferroptosis plays an important role in regulating the effects of immunotherapy. A study showed that Ubiquitin-Specific Peptidase 8(USP8) interacts with GPX4 and mediates its deubiquitination, leading to GPX4 stabilization. Consequently, USP8 inhibition destabilizes GPX4 and sensitizes cancer cells to ferroptosis *in vitro*. The combination of USP8 inhibition with ferroptosis inducers delayed tumor growth and enhanced CD8^+^ T cell infiltration, which indicated the tumor response to anti-PD-1 immunotherapy *in vivo (* 108). Thus, targeting the transporter XC^-^/GSH/GPX4 pathway combined with the use of an anti-PD-1/PD-L1 antibody has been reported as a promising therapeutic strategy for cancer. However, a recent study showed that treatment with PD-L1 inhibitor not only inhibited the activity of transporter XC^-^ in melanoma cells but also induced the polarization of macrophages to the M2 phenotype by increasing the production of PD-like exocrine 1, which leads to resistance to PD-L1 inhibitors (109). Mice bearing subcutaneous B16F10 melanoma tumors (n=5 per group) were treated with a PD-L1 inhibitor combined with sulfasalazine via intraperitoneal injection every three days and lipid peroxidation levels were not directly assessed. This discrepancy may stem from several factors. Tumor-Type Dependencies and Microenvironmental Regulation: The transporter XC⁻/GSH/GPX4 axis exhibits tissue-specific regulation across cancers. For instance, melanomas, driven by hyperactive MAPK signaling (e.g., BRAF mutations), often show constitutive PD-L1 upregulation independent of IFN-γ, predisposing them to exosomal PD-L1-mediated resistance. In EGFR-mutant lung adenocarcinoma, PD-L1 promotes proliferation and autophagy via MAPK signaling, leading to poor clinical outcomes (110). Conversely, in ovarian cancer, IFN-γ from T cells upregulates PD-L1 while downregulating SLC7A11, sensitizing cells to ferroptosis (111). Metabolic heterogeneity further shapes responses: tumors with high cysteine dependency or elevated baseline ROS may favor ferroptosis upon PD-L1 blockade. Temporal Dynamics of PD-L1 Blockade: Early PD-L1 inhibition transiently induces ferroptosis, releasing DAMPs to activate dendritic cells, but prolonged treatment selects for resistant clones secreting PD-L1-rich exosomes, driving M2 macrophage polarization. This highlights the need for clinical monitoring of treatment timing. Microenvironment Crosstalk and Clinical Implications: Stromal cells (e.g., fibroblasts) further complicate the landscape by secreting cytokines that modulate macrophage polarization independently of PD-L1 (112). Future studies must leverage multi-omics and patient-derived models to dissect tumor-specific mechanisms and optimize precision therapies. In addition, Cai verified the anti-tumor effects of statins in non-small cell lung cancer (NSCLC) (113). Specifically, statins can inhibit the expression of PD-L1 at the transcriptional level, thereby contributing to the formation of a pro-inflammatory TME and enhancing the efficacy of anti-PD-1 therapy in NSCLC. BALB/c mice subcutaneously inoculated with A549 cells, oral gavage of 40 mg/kg lovastatin combined with intraperitoneal administration of 200 μg per dose of PD-L1 antibody resulted in downregulation of GPX4 expression and increased lipid peroxidation levels in the tumor tissue.

High CD36 expression impairs PD-1 efficacy through multiple mechanisms: In T cells, CD36-mediated lipid peroxidation triggers ferroptosis, while PD-1 signaling inhibits AKT-mTOR pathway to downregulate phospholipid phosphatase 1(PLPP1), exacerbating lipid accumulation and forming a vicious cycle of “lipid metabolism-ferroptosis-immune suppression”; In ovarian/gastric cancers, CD36-high tumor cells competitively consume lipid resources in the TME, starving T cells and inducing ferroptosis to evade immune surveillance; CD36 upregulates PD-L1 via peroxisome proliferator-activated receptor gamma (PPARγ) signaling, creating dual resistance through “lipid metabolism-immune checkpoint” crosstalk (114).Targeting this pathway (via CD36 blockade or nano-delivery) can restore T cell function and overcome resistance. Future studies should develop personalized combination therapies guided by multi-omics analysis.

However, immunotherapy has both advantages and disadvantages. On one hand, it enhances anti-tumor immunity by modulating CD8^+^ T cells through the aforementioned mechanisms. On the other hand, tumor cells suppress Akt signaling in CD8^+^ T cells via the PD-L1/PD-1 axis, promoting GATA1 nuclear translocation and downregulating PLPP1 expression. This leads to the accumulation of unsaturated fatty acids, ultimately triggering ferroptosis in CD8^+^ T cells (115).

This paradox may stem from spatiotemporal differences in metabolic competition and oxidative stress. Wang’s team demonstrated that both human and murine CD4^+^ and CD8^+^ T cells exhibit resistance to ferroptosis, and Ferrostatin-1 does not affect T cell survival (34), suggesting distinct susceptibility to ferroptosis induction between tumor cells and CD8^+^ T cells. We will further elaborate on this aspect in the conclusions.

Additionally, we can identify potential biomarkers for patient stratification. For instance, Yi et al. demonstrated that the proportion of CD36^+^CD8^+^ T cells in the TME is closely associated with patient prognosis. Melanoma patients with longer survival showed lower CD36 expression on tumor-infiltrating CD8^+^ T cells compared to those with shorter survival. In bone marrow and peripheral blood samples from multiple myeloma patients, CD36 was found to be highly expressed on tumor-infiltrating CD8^+^ T cells (73).Furthermore, Tang et al. discovered that the ferroptosis inducer N6F11 specifically triggers GPX4 degradation in cancer cells without affecting GPX4 levels in immune cells (116). Studies have shown that low SLC3A2 expression is associated with average survival rate in patients (34).

Notably, ferroptosis may exacerbate immune-related adverse effects. Through lipid peroxidation and dysregulation of antioxidant systems, ferroptosis contributes to the pathogenesis of various autoimmune diseases. For example: In inflammatory bowel disease (IBD), PUFA accumulation increases intestinal epithelial cell susceptibility to ferroptosis. In rheumatoid arthritis (RA), neutrophils oxidative stress exacerbates joint damage. In systemic lupus erythematosus (SLE), ferroptosis of neutrophils promotes renal and cutaneous lesions. Targeted strategies can be employed to mitigate immune-related side effects of ferroptosis. Antibody-targeted approaches using anti-CD36/PD-L1 nanoparticles precisely act on tumor or immunosuppressive cells while avoiding damage to immune cells. Microenvironment-responsive designs enable pH/ROS-triggered nanocarriers for localized drug release, thereby reducing systemic toxicity. Antioxidant protection can be achieved by delivering Ferrostatin-1 or vitamin E to normal tissues (117). Furthermore, spatiotemporal control through light/ultrasound-triggered release allows selective protection of immune cells.

### 4.2 Ferroptosis in combination with nanomaterials

As mentioned in Section 3.1.4.2, nanomaterials such as ferumoxytol and DFHC can enhance ferroptosis by targeting iron metabolism of macrophages. This section will systematically categorize the design mechanisms of these nanomaterials and analyze their relationship with immunomodulation. Advances in cancer ferroptosis and nanotechnology have propelled the design of both iron-based and non-ferrous nanomaterials for ferroptosis regulation as a major research frontier. (**Table 2** **).**

**Table 2:** Examples of ferroptosis-modulating nanomaterials categorized by material type and mechanism.

Nanoparticle	Material type	Encapsulation	Mechanism	Reference	
FeGd-HN@Pt@LF/RGD2	IONPS	Fe3O4, Gd2O3	Promote Fenton reaction	(118)
Mn3[Co(CN)6]_2_@MIL-100 (Fe)	MOFS	Artesunate, Fe^3+^	Promote Fenton reaction	(119)
FePt@MnO@DSPE-PEG5000-FA	FePt	Fe^2+^, Mn^2+^, Folic acid	Promote Fenton reaction and Increase ROS	(120)
LDL-DHA	Self-assembled nanoparticles	low-density, lipoprotein, docosahexaenoic acid	GSH depletion, GPX4 inhibition and Lipid peroxidation	(121)
MMSNs	Self-assembled nanoparticles	Mn^2+^, SiO_2_	GSH exhaustion and ROS overloading	(122)
bcc-USINPs	Single-Crystal Fe Nanoparticle	Fe^2+^	Fe^2+^ increasing and Promote Fenton reaction	(123)
Carbon Nanoparticles−Fe (II)	Self-assembled nanoparticles	Fe^2+^	ROS overgeneration, Phase I clinical trial/NCT06048367	(124)
SRF@FeIIITA (SFT)	Self-assembled nanoparticles	Fe^3+^, TA, Sorafenib	LPO increasing	(125)
Silica nanoparticles	Self-assembled nanoparticles	Poly (ethylene glycol), peptides	ROS overgeneration	(126)
Erastin@FA-exo	Exosome	Erastin	GPX4 inhibition, CDO1 increasing and ROS overloading	(127)
TCLMS	Self-assembled nanoparticles	Lactose, Triapine, Ce6	Promote Fenton reaction, Increase chemophotodynamic therapy and Increase ROS	(128)
RF@LA-Fe-MOF	Self-assembled nanoparticles	Fe, lactobionic acid, RSL3 and iFSP1	GPX4 and FSP1 inhibition, Lipid peroxidation	(129)

IONPS, Iron oxide nanoparticles; MOFS, metal-organic frameworks; LPO: Lipid Hydroperoxide; ROS, reactive oxygenspecies; GSH, Glutathione; GPX4, Glutathione peroxidase 4; CDO1, cysteine dioxygenase 1; Ce6, Chlorin e6; FSP1, Ferroptosis suppressor protein 1; iFSP1, inhibitor of ferroptosis suppressor protein 1.

#### 4.2.1 Iron-based nanomaterials

Iron-based nanomaterials account for a large proportion of nanoscale ferroptosis inducers, as iron itself is the key component of ROS produced by Fenton reaction and the process of ferroptosis depends on iron.

Iron oxide nanoparticles (IONPs) are key iron-based nanomaterials capable of inducing tumor ferroptosis (130). Shen’s team fabricated FeGd-HN@Pt@LF/RGD2 nanoparticles that crossed the blood-brain barrier via lactoferrin receptor-mediated transport and entered cancer cells through integrin endocytosis (118). These nanoparticles released Fe²^+^/Fe³^+^ for direct Fenton reactions and cisplatin-derived H_2_O_2_ for enhanced ROS generation, while enabling MRI-guided treatment monitoring. Wang’s team created Mn3[Co(CN)6]2@MIL-100(Fe) to co-deliver artesunate and Fe³^+^, where Fe²^+^ from Fenton reactions triggered ferroptosis via ROS accumulation (119). Yang’s team constructed an acid-sensitive nano-diagnostic agent (named FePt@MnO@DSPE-PEG5000-FA, [FMDF NPs]) to monitor the response of tumor cells to ferroptosis and chemotherapy via MRI (120). FA receptor-targeted nanocomposites enabled tumor-specific Fe²^+^ delivery, triggering Fenton-driven ROS generation from H_2_O_2_ to induce ferroptosis, while Mn²^+^ enhanced MRI contrast for real-time tumor localization. Sun’s team synthesized bcc-USINPs via pyrolysis to enhance Fenton reactions and intracellular Fe²^+^ cycling for ferroptosis induction (123). SFT nanoparticles were created by coating SRF with Fe³^+^/TA complexes, enabling tumor-selective ferroptosis (125). These nanoparticles selectively caused tumor cells ferroptosis and had minimal toxic effects on normal cells. Ascorbate combined with Fe³^+^/RSL3-loaded CaP nanocarriers synergistically increased tumor-specific H_2_O_2_ and GPX4 inhibition for ferroptosis (131). FA-targeted exosomes (erastin@FA-exo) improved erastin delivery to TNBC cells, depleting glutathione and elevating ROS to promote ferroptosis while reducing systemic toxicity (127).

#### 4.2.2 Non-ferrous nanomaterials

While iron-free nanomaterials show biomedical potential, their application in ferroptosis-targeted cancer therapy remains limited due to iron’s central role in this process.

Kim et al. engineered sub-10 nm peptide/PEG-modified SiO_2_ nanoparticles that triggered ferroptosis in nutrient-deprived cancer cells and murine tumors, validating their therapeutic efficacy (126). Ou’s LDL-DHA nanoparticles selectively induced hepatoma cell death and suppressed liver tumors via iron-dependent lipid peroxidation, GSH depletion, and GPX4 inactivation in cellular/animal models (121). Leveraging Mn-O bonds’ redox sensitivity to GSH depletion (consuming 2 GSH per bond) (132), Tang’s MMSNs depleted intracellular GSH to drive hepatocellular carcinoma ferroptosis (122).

#### 4.2.3 Previous work of our team

Our team has advanced tumor ferroptosis-regulation nanomaterials, mirroring current research trends. We pioneered lactose-modified TCLM nanomicelles co-delivering Triapine/Ce6 as the first nano-DDS for Triapine-based therapy (128). Additionally, we engineered liver-targeting RF@LA-Fe-MOF nanoparticles integrating RSL3 and iFSP1 for precision ferroptosis modulation (129). Our team’s nanomaterials significantly reduce off-target toxicity through precise drug delivery. Compared to non-targeted nanomaterials, we’re the first to systematically achieve synergistic ferroptosis induction and immune microenvironment remodeling. Our advantages include: asialoglycoprotein receptor (ASGPR)-mediated efficient hepatic uptake, pH-responsive drug release for enhanced tumor accumulation, dual-target inhibition (GPX4 and FSP1) to overcome single-pathway resistance, and synergistic chemo-photodynamic therapy with Triapine and Ce6. While current ferroptosis-inducing nanomaterials show preclinical promise in animal models, their clinical translation requires rigorous validation to bridge the experimental-therapeutic gap.

We summarized common design principles emerging from research. In the field of targeted delivery, ligands such as lactobionic acid, folic acid, and apolipoproteins or targeting antibodies (e.g., PD-L1) have been utilized, thereby enhancing antitumor efficacy, improving tumor accumulation, and reducing off-target toxicity. Regarding stimuli-responsive strategies, leveraging TME features such as low pH, high GSH, and high ROS, Wang’s team constructed a dual tumor-feature (ATP and acidity) unlocked nanoplatform to amplify ferroptotic damage for efficient ferroptosis-based therapy (133). Zhang’s group reported a dual GSH/GPX4 depletion strategy using GSH-responsive polydopamine-based hybrid nanoparticles (CACuPDA) to induce tumor ferroptosis (134). Additionally, studies have identified the immune-enhancing properties of modulating TME ROS levels, guiding the selection of nanocarrier materials for nanodrug construction (135). For Fenton catalysis enhancement, iron-based nanoparticles (118)or manganese-based materials (e.g., MnO_2_) have been integrated to promote •OH generation (120, 122). In terms of GPX4 inhibition, GPX4 inhibitors (e.g., RSL3, ML162) have been delivered (127, 129), or SLC7A11 has been modulated to promote tumor cell ferroptosis (125). For combination therapies, photothermal/photodynamic therapies (e.g., Ce6-loaded systems) (128)or chemotherapeutic drugs (e.g., cisplatin) have been combined for synergistic antitumor effects (136).

However, clinical translation faces several challenges: difficulties in large-scale production of complex nanostructures requiring precise control of layered compositions (118), systemic oxidative damage (e.g., hepatotoxicity) caused by free iron ions (137), long-term retention issues of certain metal nanoparticles (138), potential complement system activation or inflammatory responses triggered by nanoparticles (139) and limited delivery efficiency due to tumor stromal barriers and immunosuppressive TME (140). To address these issues, synthesis processes can be simplified by using liposomes or polymeric micelles (e.g., PLGA) instead of complex metal nanoparticles to reduce costs, organ targeting can be optimized to mitigate side effects from specific accumulation, biodegradable materials can be employed, surface modifications of nanomaterials can minimize antibody recognition, and co-delivery of hyaluronidase or collagenase can enhance penetration depth combined with PD-1 inhibitors to reverse immunosuppression.

### 4.3 Ongoing clinical trials

Current clinical trials investigating ferroptosis in oncology remain limited in number and are predominantly in early-phase development, such as NCT06218524, phase II clinical trial, based on the Shi’s team previous work (106). Combined haloperidol and temozolomide (TMZ) may effectively treat glioblastoma by simultaneously targeting tumor cells and blocking TMZ-induced chemoresistance. The clinical data also proved that the dopamine D2 receptor (DRD2) expression in recurrent glioblastoma is significantly higher than that in primary glioblastoma. Haloperidol triggers dose-dependent ferroptosis (*in vivo*/*in vitro*), mirroring ferrostatin-1’s lipid peroxidation inhibition that attenuates its cell death effects. In addition, NCT06048367, evaluating the safety and efficacy of carbon nanoparticle-loaded iron [CNSI-Fe(II)] in advanced solid tumors (particularly Kras-mutant types, Phase I) based on Xie’s team previous work, is progressing in an orderly, systematic manner. The study also aims to evaluate the dose-limiting toxicity (DLT) of CNSI-Fe(II) to determine the maximum tolerated dose (MTD) or highest injectable dose in humans, providing dosing guidance for future clinical research (124). NCT04205357, is exploring that sulfasalazine enhances the radiotherapeutic efficacy against glioma by targeting transporter XC ⁻ to induce GSH depletion and ROS accumulation. Developing new strategies to selectively enhance radiation’s effects on tumor cells without increasing radiation-induced damage to normal brain tissue would be valuable. A newly identified NCT05924074 analyze ferroptosis levels in SF3B1-mutated myelodysplastic syndrome patients. During diagnosis, additional bone marrow samples will be aspirated. Ferroptosis will be analyzed by flow cytometry using C11-BODIPY to label lipid peroxides. Several preclinical models have demonstrated ferroptosis-enhanced antitumor immunity, such as GPX4 knockout mouse models, validating that the ferroptosis of Treg cells enhances anti-tumor immunity (141, 142). Alternatively, future studies could integrate patient-derived tumor tissues with immune cells (e.g., PDX models) to evaluate the synergistic effects of ferroptosis inducers (such as RSL3) with immune checkpoint inhibitors, as well as establish tumor organoid-immune cell (e.g., CAR-T) co-culture systems to quantitatively assess the impact of ferroptosis on immune cell infiltration and cytotoxic function.

Current clinical trials in ferroptosis research encounter multiple significant challenges. The absence of reliable ferroptosis-specific biomarkers complicates patient stratification, introducing substantial experimental variability. Methodological limitations persist, as conventional lipid peroxidation detection techniques such as fluorescent probes prove inadequate for tissue analysis or *in vivo* studies. Researchers must also contend with pronounced cell-type-dependent variations in ferroptosis susceptibility. A particularly troubling translational gap exists between *in vitro* and *in vivo* systems, where ferroptosis inhibitors demonstrating complete efficacy in cell cultures show inconsistent performance in animal models, largely due to pharmacokinetic variables and tissue-specific distribution patterns. Furthermore, interpatient response heterogeneity and potential adverse effects necessitate rigorous clinical assessment to establish therapeutic safety profiles. Although the number of clinical trials is small, the induction of ferroptosis in tumor cells in conjunction with immunotherapy and nanomaterials represents a promising strategy to overcome these challenges.

## 5 Concluding remarks and future perspectives

Ferroptosis is a recently discovered iron-dependent programmed cell death mode. In this review, we further summarized the ferroptosis of innate and adaptive immune cells in the TME and discussed key genes regulating the sensitivity of these cells to ferroptosis. In recent years, traditional anti-cancer therapies, such as chemotherapy and radiotherapy, are not always effective owing to the wide range of targets and tumor cell resistance. Compared with these therapies, immunotherapy has the advantages of remarkable curative effects, long-lasting effects and fewer side effects. Ferroptosis has been shown to enhance the anti-tumor effects of immune cells, inhibit tumor growth, prolong the survival time of patients and improve the quality of life. Meanwhile, the combined application of ferroptosis and nanomaterials is currently a research hotspot, nanomaterials have both promising therapeutic avenues and major challenges. Moreover, clinical trials investigating the therapeutic potential of ferroptosis regulators in cancer are underway (NCT06048367, NCT06218524, NCT04205357, and NCT05924074). Findings from the initial phases of both trials have validated that ferroptosis plays a major role in anti-cancer therapy. However, ferroptosis serves as a double-edged sword in cancer by both promoting and inhibiting anti-cancer immunity.

The central challenge in harnessing ferroptosis for immunotherapy lies in achieving cellular selectivity within the TME. Current strategies often lack specificity, risking collateral damage to immunostimulatory cells. Future work should focus on: Biomarker-guided targeting: Studies indicate that activated lymphocytes exhibit higher expression of TfR1 (143). This suggests that biomarker-directed drug delivery could enable precise therapeutic intervention. Exploiting metabolic reprogramming differences: Immunosuppressive cells, such as M2 macrophages, rely on glutaminase (GLS) to maintain antioxidant capacity. GLS inhibitors can selectively induce ferroptosis in M2 macrophage. Additionally, M2 macrophages depend on mitochondrial metabolism, whereas immunostimulatory cells (M1 macrophages, DCs, T cells, B cells, and NK cells) primarily utilize glycolysis for functional activation (144). Targeting these distinct metabolic pathways may allow selective ferroptosis induction. In addition, Shi and Hu team developed iron/aluminum-layered double hydroxide (Fe/Al-LDH) nanodrugs that degrade selectively in acidic microenvironments, inducing tumor cell ferroptosis while simultaneously promoting M1 macrophages polarization. This dual action synergistically enhances T-cell immunity and reverses immunosuppressive microenvironments (145).Modulating intercellular communication: Gastric cancer-derived exosomal let-7g-5p, mediated by SERPINE1, promotes M2 macrophage polarization and tumor progression (146). Conversely, exosomes like Exo@MnIO&BG, with Mn²^+^ assistance, activate the cGAS-STING pathway, inducing dendritic cell maturation and enhancing antitumor immunity (147). Thus, targeting exosomes represents a promising strategy for reshaping the TME. Epigenetic regulation: The epitranscriptional factor PCIF1 modulates m6Am modification, influencing ferroptosis-suppressing genes and the expression of CD8^+^ T cell activation markers (CD69). This regulatory mechanism negatively impacts CD8^+^ T cell activation, making them more susceptible to ferroptosis (148). Future studies should explore epigenetic approaches to selectively induce ferroptosis in immunosuppressive versus immunostimulatory cells. Temporal control strategies: Synchronizing ferroptosis induction with immune checkpoint therapy could leverage the transiently enhanced ferroptosis defense in activated CD8^+^ T cells. Combining cystine deprivation with anti-PD-L1 treatment could synergistically promote CD8^+^ T cell infiltration and establish a positive feedback loop to amplify antitumor immunity. Recent studies suggest that traditional non-selective ferroptosis induction strategies need to evolve towards more precise approaches. We propose developing a ‘Ferroptosis Immunomodulation Index’ (FII) scoring system based on GPX4/FSP1/CD36 expression profiles to better quantify the therapeutic window. A study employed the optogenetic tool Opto-GPX4Deg to demonstrate that ferroptosis can propagate to neighboring cells through α-catenin-dependent cell membrane contact, a process driven by iron-mediated lipid peroxidation and inhibitable by either α-catenin depletion or iron chelators. Liposome experiments further confirmed that lipid components alone are sufficient to mediate this transmission. These findings provide novel insights for the treatment of ferroptosis-related diseases and advance both mechanistic research and therapeutic development (149).

This review is the first to systematically delineate the differential ferroptosis responses among immune cell subsets, revealing their dual role in antitumor immunity. By innovatively proposing the “Ferroptosis Immunomodulation Index (FII)”, we provide a novel benchmark for precision cancer therapy. Beyond deciphering the metabolic and signaling networks of immune cell ferroptosis, we introduced a groundbreaking “biomarker-guided, cell-selective modulation” strategy, bridging the gap between mechanism and clinical translation. This framework, integrating theoretical depth and therapeutic innovation, offers a transformative perspective for the combined application with immunotherapy and nanomaterials.

Future directions should focus on developing humanized models (e.g., organoid-immune cocultures), combination strategies to accelerate clinical implementation. Create targeted ferroptosis therapies through three key approaches: first, by designing cell-specific inducers with advanced delivery platforms to achieve precise tumor targeting; second, by discovering definitive ferroptosis biomarkers and developing dynamic imaging modalities for accurate disease monitoring; finally, by addressing therapeutic resistance pathways including FSP1-mediated defense mechanisms in malignant cells to maintain long-term treatment effectiveness. Together, these strategic approaches will bridge the gap between ferroptosis research and clinical translation, ultimately enabling precision cancer therapies that selectively exploit this vulnerability while overcoming treatment barriers.
